# Clinical and microbiological characteristics and prognosis of invasive infection caused by *Klebsiella pneumoniae* in the community

**DOI:** 10.1080/07853890.2025.2450526

**Published:** 2025-01-10

**Authors:** Hongkui Sun, Hanlin Zhang, Wenqing Lai, Li Lei, Jianwei Li, Miaolian Chen, Haijun Li, Zhanyuan Zhao

**Affiliations:** a Critical care department, Zhongshan City People’s Hospital, Guangdong Province, China; b General Medicine Department, Zhongshan Minzhong Hospital, Guangdong Province, China; c Department of respiratory medicine, Zhongshan City People’s Hospital, Guangdong Province, China; d Department of Reproductive Medicine, Zhongshan Boai Hospital, Guangdong Province, China

**Keywords:** Invasive infection, *Klebsiella pneumoniae*, mortality, multiple drug resistance, prognosis

## Abstract

**Background:**

The incidence of invasive infection of *Klebsiella pneumoniae* (Kp) in the community is increasing every year, and the high disability and mortality rates associated with them pose great challenges in clinical practice. This study aimed to explore the clinical and microbiological characteristics of Kp invasive infection in the community.

**Method:**

This study investigated the data of 291 patients with Kp infection in the community in three hospitals (Zhongshan City, Guangdong Province) from January 2020 to August 2023. The risk factors for invasive infection and death due to Kp infection were determined through multivariate logistic regression models and Cox models.

**Result:**

The mortality rate of community-acquired *Klebsiella pneumoniae* (cKp) invasive infections markedly exceeded that for non-invasive infections (47.6% vs 25.9%) (*p* = 0.001). Multivariate logistic regression analysis identified high viscosity type (OR:2.26, *p* = 0.031) and shock (OR:3.42, *p* = 0.001) as significant risk factors for invasive infection. Among patients who succumbed to invasive infections, multivariate Cox regression analysis revealed that elevated CK-MB (OR: 1.01, *p* = 0.040), increased IL-6 levels (OR: 1.00, *p* = 0.023), and high SOFA scores (OR: 1.16, *p* = 0.017) were linked to increased mortality risk. This study found that co-infection of the liver, lungs, and bloodstream was most prevalent in invasive infections. Notably, co-infection involving the lungs, bloodstream, and brain was associated with the highest mortality rate (100%, 6/6). No significant differences were found between patients with or without invasive infections, as well as between surviving and non-surviving patients (all *p* ≥ 0.05).

**Conclusion:**

Patients with cKp invasive infections exhibit more severe inflammatory responses and a poorer prognosis, necessitating vigilant attention from clinicians. The treatment of cKp invasive infections remains inconclusive between "heavy-handed strikes" and "sensitivity is sufficient". Focusing solely on the liver and lungs while neglecting infection sites outside of these organs can lead to catastrophic results, which should be avoided during treatment.

## Introduction

*Klebsiella pneumoniae* (Kp) is an opportunistic pathogen among community-dwelling adults. As an important pathogenic bacterium in the Enterobacteriaceae family, it is one of the common pathogenic bacteria in community infections [1]. It often leads to liver abscess, pneumonia, urinary system infection, skin and soft tissue infection, blood flow infection and other infectious diseases. Clinically, 12% to 20% of patients present with coexisting infections at multiple sites [2,3], commonly classified as invasive infections [4]. The management of cKp invasive infection is challenging, with a high mortality rate [5]. These infections predominantly occur in Asian countries (The incidence rate in China is 44.1%, Vietnam is 41.3%, India is 15.2%, and Saudi Arabia is 9.6%) and are infrequent in Europe and America (Germany is 4.6%, and America is 6.3%) [6–9]. Alarmingly, their incidence is rising annually [10], leading to increased global attention towards cKp.

The most prevalent form of cKp invasive infection is a liver abscess accompanied by metastatic infections affecting other tissues or organs, such as brain abscesses, purulent meningitis, endophthalmitis, and necrotizing fasciitis [11]. Nevertheless, other infection types are being reported with increasing frequency [12]. The high disability and mortality rates associated with invasive infection of cKp pose a great threat to the human community [13,14]. In the light of this situation, numerous scholars have conducted in-depth research on the pathogen [5,10,12]. Including comparison between cKp and hypervirulent, virulent, ultravirulent and supervirulent phenotypes, in order to enable earlier identification and response [15]. At the same time, relevant vaccine research is also underway. It should be noted that although some progress has been made, the complex phenotypes and genetic characteristics of the pathogen have prevented final breakthroughs [16]. It is worth noting that the World Health Organization has recently listed Kp as a priority pathogen requiring new treatment methods [16]. Therefore, understanding its clinical and microbiological characteristics is of great significance for physicians to better respond to infections caused by this pathogenic bacterium. But the scarcity of relevant clinical studies, the clinical features of cKp invasive infections remain poorly defined. Notably, the striking disparity between favorable drug sensitivity results and the poor prognosis associated with these infections is troubling and presents significant challenges in clinical management. Hence, we aimed to elucidate the clinical and microbiological characteristics of cKp invasive infection in Zhongshan, Guangdong, China. To date, no studies on this topic have been published.

## Materials and methods

### Patients

**Figure 1.**
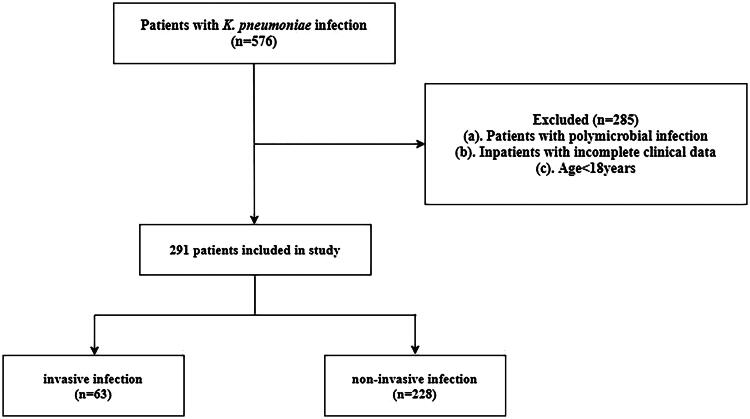
Flow chart of this study.

We retrospectively analysed the data of patients with cKp infection from Zhongshan City People’s Hospital (Three Level of First-class Hospital. The number of outpatient and emergency patients is about 3 million per year, with more than 90,000 discharges per year), Zhongshan Minzhong Hospital (Secondary-level Hospital. The outpatient volume is about 300,000 per year, with over 10,000 discharges per year) and Zhongshan Boai Hospital (Three Level of First-class Hospital. The number of outpatient and emergency patients is about 1.93 million per year, with about 50,000 discharges per year). The study period extended from January 2020 to August 2023. During this period, we collected data on 597 patients with cKp infection, 291 of whom were finally enrolled (Figure 1). Kp was detected in the pathogen culture (blood, pus, clean midstream urine, bronchoalveolar lavage fluid and sputum, cerebrospinal fluid) of all patients within 48 h of admission. This has been determined to have clinical significance. Patients were categorized into invasive and non-invasive groups based on their infection status at the time of admission. CKp invasive infection is defined as the simultaneous involvement of two or more organs or sites at admission, excluding bloodstream infections. Diagnosis of endophthalmitis relies on symptoms such as reduced visual acuity, eye pain, anterior chamber pus, or severe anterior uveitis, alongside bacteraemia [17]. Liver abscess diagnosed through the presence of fever, hepatic pain, and the detection of a low-density mass on ultrasonography or computed tomography, characterized by annular bands of varying densities. Contrast-enhanced scans may reveal an unchanged cavity density, irregular thickening of the cavity wall, and Kp identified in pus culture [17]. Soft tissue infection involves the subcutaneous tissue, fascia, or muscle [18]. Intracranial infection is diagnosed based on fever, altered consciousness, and the detection of Kp in cerebrospinal fluid culture [19]. Urinary tract infection is confirmed by an elevated white blood cell count in urine and a bacterial colony count of ≥10^5^/ml in a clean-catch urine culture [20].

In these patients, third-generation cephalosporin, imipenem (IPM)/cilastatin, meropenem, moxifloxacin, piperacillin, tazobactam, levofloxacin (LVX) were administered as first-line antibiotics for infection control. Clinicians decided whether to use single or dual drugs to control the infection based on the individual situation. We analysed the patient’s age, sex, underlying disease, infection site, anti-infection regimen, microbiological characteristics, laboratory tests and SOFA scores during admission, and other data. This study obtained written informed consent from the participants. This study was approved by the Ethics Committee of Zhongshan City People’s Hospital (2024-001) and complies with the Declaration of Helsinki.

### Inclusion criteria

The inclusion criteria were availability of complete data and Kp infection acquired from the community. The pathogenic specimens analysed included blood, pus, clean midstream urine, bronchoalveolar lavage fluid, sputum and cerebrospinal fluid from patients admitted within 48 h, and Kp was the identified pathogen.

### Exclusion criteria

The exclusion criteria were age <18 years and infection with multiple pathogenic microorganisms.

### Definition

Multidrug-resistant (MDR) strains refer to bacteria exhibiting simultaneous resistance to three or more types of antibiotics [21].

### Data collection

Patient data were collected through the hospital information system and inclusion/exclusion criteria applied. Demographic information, infection site, microbiological, and other outcome data were collected during hospitalization.

### Pathogen analysis

Microscan-walk-way 96 plus (Siemens, Germany) was used for microbial identification and drug sensitivity testing. The drug sensitivity results were determined according to the 2016 US Clinical and Laboratory Standards Institute guidelines [22]. All pathogenic bacteria identified were rechecked using the matrix-assisted laser desorption ionization time-of-flight mass spectrometer.

### Statistical analysis

Continuous variables are presented as medians and interquartile ranges (IQR) (PR25, PR75). Inter-group comparisons were conducted using the Mann–Whitney U-test. Categorical variables are presented as number and percentage, and they were compared using Chi-square test or Fisher’s exact text (if expected value ≤ 5 was found). Multivariate logistic regression model and Cox proportional-hazards model were used to investigate the independent variables associated with the risk of invasive infection and overall survival, respectively. The independent variables that were significant in inter-group comparisons were used in the multivariate model analysis (forward method). The independent variables that were significant in the multivariate logistic and Cox model analyses were recognized as factors associated with invasive infection and overall survival, respectively. The receiver operating characteristics (ROC) analysis was used to investigate the diagnostic effectiveness of both multivariate logistic and Cox models for invasive infection and overall survival by analyzing the estimated risk probabilities and hazard functions. All analyses were performed using Statistical Product and Service Solutions, version 25 (IBM Corporation, Somers, New York). Two-tailed *P*-values <0.05 were considered statistically significant for all the tests. The diagnostic effectiveness of this multivariate model was checked by conducting an ROC analysis using statistical software R (version 4.0.5) and the package ‘pROC’. The log-rank test was conducted using the package ‘survminer’.

## Results

### Invasive infection characteristics of Klebsiella pneumoniae

This study included 291 patients, consisting of 215 men (73.9%) and 76 women (26.1%) (Table 1). Among these, 63 cases (21.6%) were classified as invasive infections and 228 cases (78.4%) as non-invasive infections. There were no significant differences in average age or gender ratio between two groups (*p* > 0.05). Notably, 81.4% of patients with invasive infections had underlying conditions, with diabetes being the most prevalent (50.8%) (Table 1). Chronic kidney disease was significantly associated with a higher likelihood of invasive infections compared to non-invasive infections (*p* = 0.042) (Table 1). Patients with invasive infections exhibited elevated levels of PCT (*p* = 0.018), IL-6 (*p* = 0.000), BNP (*p* = 0.012), alongside reduced PLT levels (*p* = 0.044). No differences were observed in other laboratory indicators. Those with invasive infections had higher SOFA scores (*p* = 0.000), greater incidence of septic shock (*p* = 0.000), longer ICU stays (*p* = 0.000) and increased mortality rates (*p* = 0.001). Other clinical evaluations showed no significant differences. Bacterial resistance and hospital stay did not differ significantly between the groups (all *p* > 0.05).

**Table 1.** Patient’s clinical characteristics by invasive infection group.

	Invasive infection			
Parameters	Yes (*n* = 63)	No (*n* = 228)	All (*n* = 291)	*P* value
Age, year	60 (52, 74)	65(52, 73)	64 (52, 74)	0.550
Gender				0.149
Male	51 (81.0%)	164 (71.9%)	215 (73.9%)	
Female	12 (19.0%)	64 (28.1%)	76 (26.1%)	
Underlying disease	53(81.4%)	164 (71.9%)	217 (74.6%)	
Diabetes	32 (50.8%)	95 (41.7%)	127 (43.6%)	0.196
Cardiovascular disease	12 (19.0%)	28 (12.3%)	40 (13.7%)	0.167
Cerebrovascular disease	12(19.0%)	40 (17.5%)	52 (17.9%)	0.783
Chronic pulmonary disease	2 (3.2%)	15 (6.6%)	17 (5.8%)	0.542
Chronic kidney disease	10 (15.9%)	17 (7.5%)	27 (9.3%)	**0.042** *
Hepatopathy	5 (7.9%)	15 (6.6%)	20 (6.9%)	0.778
Tumor and autoimmune diseases	6 (9.5%)	28 (12.3%)	34 (11.7%)	0.547
High viscosity type	50 (79.4%)	130 (57.0%)	180 (61.9%)	**0.001** *
Laboratory result				
WBC	12.1 (6.7, 20.0)	12.7 (7.7, 16.9)	12.5 (7.4, 17.6)	0.918
PCT	30 (2, 79)	6 (2, 39)	10 (1, 42)	**0.018** *
IL-6	1121 (177, 5000)	230 (69, 890)	292 (80, 1910)	**0.000** *
PLT	114 (34, 207)	147 (95, 242)	141 (77, 242)	**0.044** *
CK	160 (33, 412)	78 (37, 288)	88 (37, 325)	0.101
CK-MB	18 (10, 27)	15 (10,23)	15 (10, 24)	0.160
BNP	1843 (768, 8854)	1139 (347, 5226)	1235 (418, 6207)	**0.012** *
Cr	115 (59,163)	94 (69,155)	97 (69, 155)	0.509
Drug-resistant strains	18 (28.6%)	63 (27.6%)	81 (27.8%)	0.883
MDROs	2 (3.2%)	25 (11.0%)	27 (9.3%)	0.059
SOFA	8 (4, 11)	4 (2, 7)	5 (2, 8)	**0.000** *
Septic shock	39 (61.9%)	54 (23.7%)	93 (32.0%)	**0.000** *
Antibiotic exposure	8 (12.7%)	32 (14.0%)	40 (13.7%)	0.785
Hospital stay, days	16 (7, 21)	15 (10, 22.75)	15 (9, 22)	0.477
ICU stay, days	6 (3, 12)	1 (0, 6)	2 (0, 7)	**0.000** *
Outcome				**0.001** *
Survival	33 (52.4%)	169 (74.1%)	202 (69.4%)	
Dead	30 (47.6%)	59 (25.9%)	89 (30.6%)	

WBC: white blood cells; PCT: procalcitonin; IL-6: interleukin-6; PLT: platelets; CK: creatine kinase; CK-MB: creatine kinase-MB isoenzyme; BNP: brain natriuretic peptide; Cr: creatinine; MDROs: multidrug-resistant organism; SOFA: sequential organ failure score; ICU: Intensive Care Unit.

*P-value ≤ 0.05 are considered significant.

### Characteristics of patients who died from cKp invasive infections

Patients with lower PLT and CK-MB levels exhibited poorer prognoses (*p* < 0.05) (Table 2). Those who died from invasive infections had significantly higher SOFA scores (*p* = 0.000), and shorter hospital stays (*p* = 0.000). Notably, no significant differences were observed in septic shock incidence or ICU stay between survivors and deceased patients (*p* > 0.05) (Table 2).

**Table 2.** Characteristics of deceased patients.

	Clinical outcome		
Parameters	Dead (*n* = 30)	Survival (*n* = 33)	*P* value
Age, year	50 (53, 75)	57 (47, 66)	0.083
Gender			0.854
Male	24 (80.0%)	27 (81.8%)	
Female	6 (20.0%)	6 (18.2%)	
Underlying disease	25 (83.3%)	28 (84.8%)	1.000
Diabetes	16 (53.3%)	16 (48.5%)	0.701
Cardiovascular disease	7 (23.3%)	5 (15.2%)	0.409
Cerebrovascular disease	6 (20.0%)	6 (18.2%)	0.854
Chronic lung disease	0 (0.0%)	2 (6.1%)	0.493
Chronic kidney disease	3 (10.0%)	7 (21.2%)	0.308
Hepatopathy	3 (10.0%)	2 (6.1%)	0.662
Tumor and autoimmune diseases	5 (16.7%)	1 (3.0%)	0.094
Laboratory result			
WBC	9.7 (5.4, 15.7)	17.0 (8.5, 20.3)	0.061
PCT	37 (6, 81)	14 (2, 74)	0.151
IL-6	2731 (134, 5000)	426 (221, 1989)	0.070
PLT	87 (14, 127)	150 (71, 298)	0.001*
CK	322 (62, 793)	136 (26, 334)	0.070
CK-MB	23 (17, 67)	13 (10, 19)	0.002*
BNP	1670 (698,9000)	1843 (783, 7516)	0.382
Cr	125 (93, 153)	83 (54, 191)	0.057
Drug-resistant strains	4 (16.7%)	14 (35.9%)	0.101
MDROs	0 (0.0%)	2 (5.1%)	0.521
SOFA	11 (8, 14)	6 (3, 8)	0.000*
Septic shock	22 (73.3%)	17 (51.5%)	0.075
Antibiotic exposure	2 (8.3%)	6 (15.4%)	0.699
Number of antibiotics			0.280
1	16 (53.3%)	22(66.7%)	
2	14 (66.7%)	11(33.3%)	
Clinical outcome			
Hospital stay, days	8 (3, 18)	18 (14,29)	0.000*
ICU stay, days	6 (3, 12)	2 (6, 10)	0.535

WBC: white blood cells; PCT: procalcitonin; IL-6: interleukin-6; PLT: platelets; CK: creatine kinase; CK-MB: creatine kinase-MB isoenzyme; BNP: brain natriuretic peptide; Cr: creatinine; MDROs: multidrug-resistant organism; SOFA: sequential organ failure score; ICU: Intensive Care Unit.

*P-value ≤ 0.05 are considered significant.

### Characteristics of infection sites (each type of infection)

**Figure 2.**
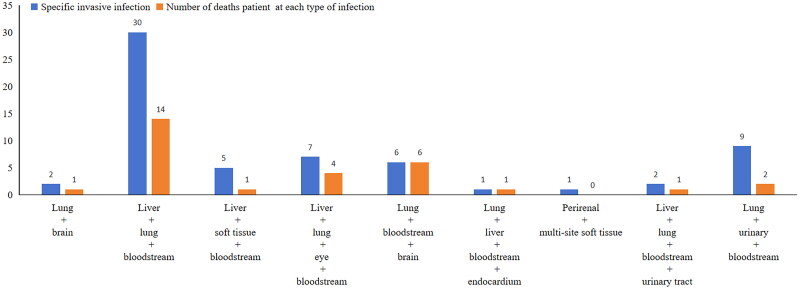
Distribution of infected patients and mortality by infection type (invasive infections).

Figure 2 illustrates that the most frequent co-infections in invasive cases involved the liver, lungs, and bloodstream, comprising 47.6% (30/63) of the cases. The highest mortality rate was associated with infections involving the lungs, bloodstream, and brain, reaching 100% (6/6). Additionally, a case involving simultaneous infection of the liver, lungs, bloodstream, and endocardium was observed, with the patient unfortunately succumbing to the infection. Overall, invasive infections exhibited a high mortality rate of 47.6% (Table 1).

### Factors related to cKp invasive infection

Table 3 presents the logistic regression model analysis for factors associated with cKp invasive infection. The analysis identifies that high viscosity type (OR: 2.26, *p* = 0.031) and shock (OR: 3.42, *p* = 0.001) are significantly associated with an increased risk of invasive infection.

**Table 3.** Factors related to invasive infections.

Parameters	Univariate analysis		Multivariate analysis	
	OR (95% CI)	P Value	OR (95% CI)	P Value
Age, year	0.99 (0.97 to 1.01)	0.366		
High viscosity type	2.89 (1.49 to 5.63)	0.002*	2.26 (1.07 to 4.74)	0.031*
Cardiovascular disease	1.68 (0.80 to 3.53)	0.171		
Diabetes	1.44 (0.82 to 2.52)	0.197		
WBC	1.00 (0.97 to 1.04)	0.811		
PCT	1.01 (1.01 to 1.02)	0.004*		
IL-6	1.00 (1.00 to 1.00)	0.002*		
PLT	0.99 (0.99 to 1.00)	0.331		
CK	1.00 (0.99 to 1.00)	0.352		
CK-MB	1.00 (0.99 to 1.01)	0.136		
BNP	1.00 (1.00 to 1.00)	0.177		
Cr	1.00 (1.00 to 1.00)	0.970		
SOFA	1.17 (1.10 to 1.26)	0.000*		
Septic shock	5.23 (2.89 to 9.45)	0.000*	3.42 (1.61 to 7.27)	0.001*

WBC: white blood cells; PCT: procalcitonin; IL-6: interleukin-6; PLT: platelets; CK: creatine kinase; CK-MB: creatine kinase-MB isoenzyme; BNP: brain natriuretic peptide; Cr: creatinine; SOFA: sequential organ failure score.

*P-value ≤ 0.05 are considered significant.

### Factors related to invasive infection mortality

Table 4 show the multivariate regression modelling results of the Cox model. High CK-MB (OR: 1.01, *p* = 0.040), IL-6 level (OR: 1.00, *p* = 0.023), and high SOFA scores (OR:1.16, *p* = 0.017) were associated with the risk of invasive infection mortality.

**Table 4.** Multivariate Cox proportional-hazard regression model results.

Parameters	Univariate analysis		Multivariate analysis	
	OR (95% CI)	*P* Value	OR (95% CI)	*P* Value
Age, year	1.02 (0.99 to 1.05)	0.079		
High viscosity type	1.54 (0.62 to 3.83)	0.351		
Cardiovascular disease	2.15 (0.91 to 5.11)	0.082		
Diabetes	0.83 (0.40 to 1.72)	0.619		
WBC	0.94 (0.89 to 0.99)	0.022		
PCT	1.01 (1.001 to 1.02)	0.117		
IL-6	1.00 (1.00 to 1.00)	**0.000** *	1.00(1.00 to 1.00)	**0.023** *
PLT	0.99 (0.99 to 1.00)	**0.009** *		
CK	1.00 (1.00 to 1.00)	**0.006** *		
CK-MB	1.01 (1.01 to 1.02)	**0.001** *	1.01 (1.01 to 1.02)	**0.040** *
BNP	1.00 (1.00 to 1.00)	0.180		
Cr	1.00 (0.99 to 1.00)	0.457		
SOFA	1.23 (1.13 to 1.34)	**0.000** *	1.16 (1.03 to 1.31)	**0.017** *
Septic shock	2.44 (1.07 to 5.35)	**0.033** *		
Number of antibiotics				
1	ref.	–		
2	1.29 (0.63 to 2.67)	0.476		

WBC: white blood cells; PCT: procalcitonin; IL-6: interleukin-6; PLT: platelets; CK: creatine kinase; CK-MB: creatine kinase-MB isoenzyme; BNP: brain natriuretic peptide; Cr: creatinine; SOFA: sequential organ failure score.

*P-value ≤ 0.05 are considered significant.

### ROC analysis and AUC

To further validate the predictive efficacy of the multivariate models, the probabilities generated by these models were evaluated using ROC analysis.

**Figure 3.**
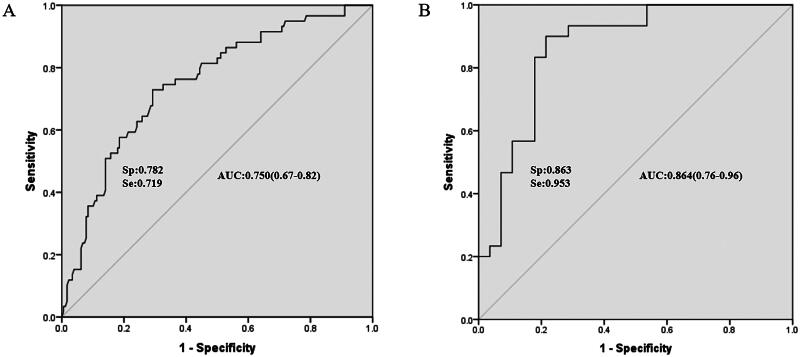
ROC analysis evaluates the predictive power of two multivariate models.

Figure 3(A and B) reveal that the logistic and Cox models exhibited moderate diagnostic performance, with areas under the ROC curve of 0.750 and 0.864, respectively.

### Microbiology

**Figure 4.**
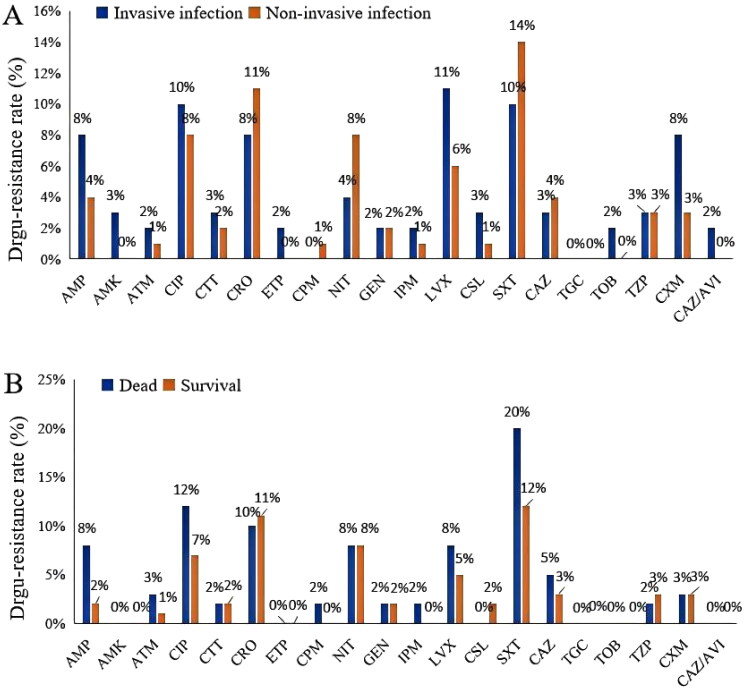
The charts of drug-resistance rates by invasive infection group (A) and clinical outcome group (B).

The following drugs were investigated for bacterial resistance in this study: ampicillin (AMP), amikacin (AMK), aztreonam (ATM), ciprofloxacin (CIP), cefotetan (CTT), cefatriaxone (CRO), ertapenem (ETP), cefepime (CPM), nitrofurantoin (NIT), gentamicin (GEN), IPM, LVX, cefperazone/sulbactam (CSL), trimethoprim-sulfamethoxazole (SXT), ceftazidime (CAZ), tigecycline (TGC), tobramycin (TOB), piperacillin/tazobactam (TZP), cefuroxime (CXM), and ceftazidime-avibactam (CAZ/AVI) (Figure 4). Antibiotic exposure was observed in 13.7% of patients (Table 1). Notably, drug-resistant strains and MDR strains accounted for 27.8% and 9.3% of all strains, respectively (Table 1). However, no significant differences were found between patients with and without invasive infections and between surviving and non-surviving patients (all *p* > 0.05) (Figure 4). The three drugs with the highest drug-resistance rates in the invasive infection group were as follows: LVX (11%), SXT (10%) and CIP (10%) (Figure 4).

## Discussion

Kp often leads to severe community-acquired infections, which are invasive and migratory. Currently, consensus on relevant treatments is lacking. The contradiction between ideal antibiotic sensitivity results and poor prognosis poses great challenges to clinical practice. Previous studies have mainly focused on high mucosal phenotype, serotype, and virulence genes. Due to the inherent nature of cKp, its characteristics of its invasive infection are still not fully understood. Moreover, previous clinical studies on cKp infection have mostly focused on a single site of infection. We analysed patients with invasive cKp infection and those who died, thus filling the current research gap.

The incidence rate of cKp invasive infections has been reported in various regions, with significant regional differences (9%∼37%) [17,23]. In our study, invasive infections accounted for 21.6%, which was slightly lower than that reported previously. This discrepancy may arise from various factors, including epidemiological variations, racial genetic susceptibility, and pathogen-specific genetic traits, though the exact mechanisms remain unclear. Previous studies have indicated that invasive infections by this pathogen are often associated with co-infections of the liver, brain, and lungs or liver, brain and eyes [17,24]. While our findings align with these reports, they also reveal that the most prevalent infection type in our cohort was the co-infection of the liver, lungs, and bloodstream (30/63). Significant correlations between pathogen serotypes and specific infection types have been observed [25], but current data are still limited, necessitating further research.

Reports suggest that invasive infections are more prevalent among younger individuals [26]. However, our study did not reveal significant age differences. Liu C et al. also found no correlation between age and invasive infections [27]. Given the specificity of the disease and sample size limitations, further confirmation through multi-center large-scale studies is needed.

Patients with cKp invasive infections typically have lower PLT and albumin levels and elevated total bilirubin [28]. Our study observed more severe inflammatory responses in these patients, with higher PCT and IL-6 levels, lower PLT, elevated SOFA scores, and a higher incidence of shock. These findings align with previous research [28]. Elwakil BH et al. reported increased IL-6 levels and severe brain tissue damage in a rat model of Kp-induced encephalitis [29]. Liu C et al. found significantly higher WBC and SOFA scores in patients with aggressive infections compared to those with non-invasive infections, though they did not examine PCT and IL-6 levels [27]. The absence of significant differences in WBC levels between our study groups may be attributed to variations in population characteristics and sample size. Additionally, patients with invasive infections had longer ICU stays and poorer prognoses, as confirmed by previous studies [30]. Univariate logistic regression analysis identified hypermucoviscous bacteria, elevated PCT and IL-6 levels, shock, and high SOFA scores as associated with cKp invasive infections. Multivariate logistic regression analysis further established hypermucoviscous phenotype and shock as independent risk factors. Conversely, Li L et al. identified diabetes as a risk factor for cKp invasive infections, attributing it to hyperglycaemia’s role in stimulating biofilm formation and enhancing bacterial resistance and invasiveness [31,32]. Lee HC et al. highlighted the association of the hypermucous phenotype with increased virulence and invasiveness of Kp [32,33]. Notably, our study is the first to identify shock as an independent risk factor for cKp invasive infection.

In our study, the mortality rate for patients with invasive infections was 47.6% (30/63), which is notably higher than previously reported rates (3-31%) [4,13,34]. This variation may be attributed to differences in regional factors, including patient population characteristics, treatment and management protocols, pathogen serotypes, and genetic factors. We observed that patients who died from invasive infections had lower PLT, elevated CK-MB levels, higher SOFA scores, and shorter hospital stays. The mechanism behind elevated CK-MB remains unclear, but may be related to septic myocardial abscesses, myocarditis or embolic myocardial infarction following Kp infections [35, 36]. Hypermucoviscous Kp is known to significantly induce platelet aggregation and apoptosis, and inhibit megakaryocyte maturation [37]. Other studies have identified additional factors, such as ICU admission and the presence of the iroN gene which is associated with enhanced pathogenicity and iron acquisition [38]. This gene has been linked to increased invasive infection ability in mouse model [33]. Our study being the first to identify risk factors for mortality in Kp invasive infections, found that univariate analysis associated lower WBC and PLT levels, higher IL-6, CK, CK-MB levels, septic shock, and high SOFA scores with increased mortality. However, multivariate Cox regression analysis identified high IL-6, CK-MB levels, and high SOFA scores as independent risk factors. Wang H et al.’s reported that mice infected with Kp had severe inflammatory responses, shorter survival times, and higher mortality [39]. Elevated CK-MB levels may result from increased inflammatory factor release, leading to cardiac insufficiency and myocardial infarction [40]. Given the limited clinical research data on cKp invasive infections, further studies are needed to validate these findings.

Anti-infective regimens play an important role in the treatment process. Currently, no standard treatment plan or consensus exists for cKp infection. In our study, third-generation cephalosporins, carbapenems, fluoroquinolones, and TZP were used as first-line anti-infective agents. Clinically, the administration of anti-infective drugs should be considered in conjunction with factors such as the site of infection, the patient’s underlying health status, organ function, and immune status. In this study, the combination therapy of two antibiotics did not reduce the mortality rate. Nonetheless, this association with mortality should be verified through prospective studies in the future. Liver, soft tissue, and bloodstream infections exhibited lower mortality rates compared to other types of infections. This may be attributed to the effectiveness of drainage and antibiotics in managing these infections.

Pathogenic antibiotic resistance has attracted global attention. Kp as one of the drug-resistant ESKAPE pathogens. It is the second leading cause of death from antibiotic-resistant pathogens worldwide and the leading cause of death in sub-Saharan Africa [41]. In our study, drug-resistant strains accounted for 27.49%, while MDR strains accounted for 11%, all produced extended-spectrum β-lactamase (ESBLs), no carbapenem-resistant strains were found. Although drug-resistant strains did not have a significant impact on treatment outcomes, they still posed challenges to clinical treatment. The spread of MDR Kp in the community requires our attention, and measures should be taken to prevent spread of drug-resistant strains. Notably, among the top five drugs with the highest resistance rates, three are commonly used in clinical practice, including CRO (8%), CIP (10%) and LVX (11%). The resistance of Kp to third-generation cephalosporins has increased, and the sensitivity to carbapenems decreased or even resistance to carbapenems in some areas. Due to drug resistance, effective treatment options are reduced, making the management of infections more difficult and imposing a significant economic burden on patients, especially in developing countries. Therefore, when managing CKp infection cases, we need to be cautious and provide appropriate treatment plans based on the patient’s condition to improve efficacy and reduce the development of drug-resistant bacteria.

Our research has limitations. Firstly, we only studied cKp infection in Zhongshan City of Guangdong, China, and this study had a small sample size. Secondly, we did not analyze the genes and serotypes of the pathogenic bacteria.

## Conclusion

Overall, cKp invasive infections are associated with a high mortality rate. High viscosity type and shock have been identified as risk factors for invasive cKp infections. Elevated CK-MB, IL-6 levels, and high SOFA scores are associated with increased mortality from cKp infections. The treatment of cKp invasive infections remains inconclusive between "heavy-handed strikes" and "sensitivity is sufficient". Focusing solely on the liver and lungs while neglecting infection sites outside of these organs can lead to catastrophic results, which should be avoided during treatment.

## Data Availability

The data for this study can be obtained from the corresponding authors upon request.
